# CNS manifestations in acute and chronic graft-versus-host disease

**DOI:** 10.1093/brain/awae340

**Published:** 2024-10-23

**Authors:** Nicolas Lambert, Florence Forte, Majdouline El Moussaoui, Justine Monseur, Nicole Raus, Alexey Polushin, David Michonneau, Carl Shultz, William J Hogan, Aitana Balaguer-Roselló, Sara Gil-Perotín, Jan Brijs, Paul Chauvet, Maria Gavriilaki, Martin Carre, Adriana Octaviana Dulamea, Yves Chalandon, Urpu Salmenniemi, Andrea Duminuco, Ron Ram, Irene García-Cadenas, Gaetana Porto, Stéphanie Nguyen, Portia Smallbone, Marta González-Vicent, Jonathan D Santoro, Evelyne Willems, Frédéric Baron, Sophie Servais, Yves Beguin, Pierre Maquet, the CNS-GvHD Study Group, Nicolas Lambert, Florence Forte, Majdouline El Moussaoui, Justine Monseur, Nicole Raus, Alexey Polushin, Iaroslav Skiba, David Michonneau, Carl Shultz, William J Hogan, Aitana Balaguer-Roselló, Sara Gil-Perotín, Jan Brijs, Paul Chauvet, Maria Gavriilaki, Ioanna Sakellari, Martin Carre, Adriana Octaviana Dulamea, Alina Daniela Tanase, Yves Chalandon, Sylvain Chantepie, Andrea Duminuco, Urpu Salmenniemi, Michael Loschi, Ron Ram, Irene Garcia Cadenas, Gaetana Porto, Massimo Martino, Patrycja Mensah-Glanowska, Sara Butera, Portia Smallbone, Agnieszka Piekarska, Jeffrey K Davies, Jonathan D Santoro, Hélène Labussière-Wallet, Marta Gonzalez Vicent, Stéphanie Nguyen, Maud D’Aveni, Mehdi Hamadani, Evelyne Willems, Frédéric Baron, Pierre Maquet, Yves Beguin, Sophie Servais

**Affiliations:** Department of Neurology, University Hospital of Liège, 4000 Liège, Belgium; Department of Neurology, University Hospital of Liège, 4000 Liège, Belgium; Department of Infectious diseases and General Internal Medicine, University Hospital of Liège, 4000 Liège, Belgium; Biostatistics and Research Methods center (B-STAT), Department of Public Health, University of Liège, 4000 Liège, Belgium; Department of Hematology Marcel-Bérard secteur 1G, Centre hospitalier Lyon sud, 69310 Pierre-Bénite, France; Department of Chemotherapy and Stem Cell Transplantation for Cancer and Autoimmune Diseases, First Pavlov State Medical University of St. Peterburg, 197022 Saint Petersburg, Russia; Hematology and Transplantation Unit, Saint Louis Hospital, Université Paris Cité, 75010 Paris, France; Division of Hematology, Mayo Clinic, Rochester, MN 55905, USA; Division of Hematology, Mayo Clinic, Rochester, MN 55905, USA; Department of Hematology, Hospital Universitari i Politècnic la Fe, 46026 Valencia, Spain; Department of Neurology, Hospital Universitari i Politècnic la Fe, 46026 Valencia, Spain; Department of Hematology, University Hospitals Leuven, 3000 Leuven, Belgium; CHU de Lille, Maladies du Sang, Université de Lille, 59000 Lille, France; First Department of Neurology, AHEPA University Hospital, Aristotle University of Thessaloniki, 54636 Thessaloniki, Greece; Department of Hematology, Hôpital Michallon, 38043 Grenoble, France; Department of Neurology, University of Medicine and Pharmacy ‘Carol Davila’, Fundeni Clinical Institute, Bucharest 022328, Romania; Division of Hematology, University Hospital of Geneva (HUG) and faculty of Medicine, University of Geneva, 1205 Geneva, Switzerland; HUCH Comprehensive Cancer Center, Stem Cell Transplantation Unit—Helsinki, 00029 Helsinki, Finland; Department of Hematology with BMT, A.O.U. Policlinico ‘G.Rodolico-San Marco’, 95123 Catania, Italy; BMT Unit, Tel Aviv Sourasky Medical Center and Faculty of Medicine, Tel Aviv University, Tel Aviv 6423906, Israel; Department of Hematology, Hospital de la Santa Creu i Sant Pau, 08025 Barcelona, Spain; Department of Hemato-Oncology and Radiotherapy, Stem Cell Transplantation and Cellular Therapies Unit (CTMO), Grande Ospedale Metropolitano ‘Bianchi-Melacrino-Morelli’, 89100 Reggio Calabria, Italy; Department of Hematology, Pitie-Salpetriere Hospital, 75013 Paris, France; Department of Hematology, Fiona Stanley Hospital, 6150 Perth, Western Australia; BMT Unit, Hospital Niño Jesus, 28009 Madrid, Spain; Division of Neurology, Department of Pediatrics, Children’s Hospital Los Angeles, Los Angeles, CA 90027, USA; Department of Hematology, University Hospital of Liège, 4000 Liège, Belgium; Department of Hematology, University Hospital of Liège, 4000 Liège, Belgium; Department of Hematology, University Hospital of Liège, 4000 Liège, Belgium; Department of Hematology, University Hospital of Liège, 4000 Liège, Belgium; Department of Neurology, University Hospital of Liège, 4000 Liège, Belgium

**Keywords:** GvHD, neurological complications, encephalitis, brain lesions, spinal cord lesions, immune-mediated

## Abstract

Despite the growing evidence supporting the existence of CNS involvement in acute and chronic graft-versus-host disease (CNS-GvHD), the characteristics and course of the disease are still largely unknown. In this multicentre retrospective study, we analysed the clinical, biological, radiological and histopathological characteristics, as well as the clinical course of 66 patients diagnosed with possible CNS-GvHD (pCNS-GvHD), selected by predetermined diagnostic criteria. Results were then contrasted depending on whether pCNS-GvHD onset occurred before or after Day 100 following allogeneic haematopoietic stem cell transplantation (allo-HSCT).

The median time between allo-HSCT and pCNS-GvHD onset was 149 days (interquartile range_25–75_ 48–321), and pCNS-GvHD onset occurred before Day 100 following transplantation in 44% of patients. The most frequent findings at presentation were cognitive impairment (41%), paresis (21%), altered consciousness (20%), sensory impairment (18%) and headache (15%). Clinical presentation did not significantly differ between patients with pCNS-GvHD occurring before or after Day 100 following transplantation.

Brain MRI found abnormalities compatible with the clinical picture in 57% of patients, while CT detected abnormalities in only 7%. Seven patients had documented spinal cord MRI abnormalities, all of them with pCNS-GvHD occurring after Day 100 following transplantation. In the CSF, the white blood cell count was increased in 56% of the population (median 18 cells/μl). Histopathological analyses were performed on 12 specimens and were suggestive of pCNS-GvHD in 10. All compatible specimens showed parenchymal and perivascular infiltration by CD3+ and CD163+ cells.

Immunosuppressive therapy was prescribed in 97% of patients, achieving complete clinical response in 27%, partial improvement in 47% and stable disease in 6%. Response to immunosuppressive therapy did not differ significantly between patients with pCNS-GvHD occurring before or after Day 100 following transplantation. Clinical relapse was observed in 31% of patients who initially responded to treatment. One-year overall survival following pCNS-GvHD onset was 41%. Onset before Day 100 following haematopoietic stem cell transplantation [hazard ratio with 95% confidence interval: 2.1 (1.0–4.5); *P* = 0.041] and altered consciousness at initial presentation [3.0 (1.3–6.7); *P* = 0.0077] were associated with a reduced 1-year overall survival probability. Among surviving patients, 61% had neurological sequelae. This study supports that immune-mediated CNS manifestations may occur following allo-HSCT.

These can be associated with both acute and chronic GvHD and carry a grim prognosis. The clinical presentation as well as the radiological and biological findings appear variable.

## Teaser

In a retrospective international multicentre study, Lambert *et al.* describe the clinical, biological, radiological, and histopathological characteristics, as well as the course and treatment response, of a large cohort of patients with CNS involvement in graft-versus-host disease.

## Introduction

Graft-versus-host-disease (GvHD) is a severe and potentially life-threatening complication of allogeneic haematopoietic stem cell transplantation (allo-HSCT).^1^ It arises when the donor’s derived immune cells recognize the recipient’s healthy tissues as ‘non-self’, thereby generating an allo-immune reaction.^2^ Its two main presentations include acute and chronic GvHD, characterized by distinct clinical manifestations and pathophysiological mechanisms.^3-5^ The CNS was initially considered protected from GvHD. Yet, following the accumulation of reports of patients with neurological manifestations for which the pathological mechanism was thought to be immune-mediated, CNS involvement in chronic GvHD was recognized as an entity in 2010 following the Consensus Conference on Clinical Practice in chronic GvHD.^6^ Based on this report, the diagnosis of ‘possible’ CNS involvement in chronic GvHD could be made in patients with classic manifestations of chronic GVHD affecting other organs (first major mandatory criterion), presenting with neurological signs of CNS involvement without other explanation (second major criterion) and at least two other minor diagnostic criteria (corresponding brain MRI abnormality, abnormal CSF studies, CNS neuropathology revealing lesions compatible with GvHD and response to immunosuppressive therapy).

Despite progress, there are still many unknowns in the field of CNS involvement in the context of GvHD (CNS-GvHD). Because only isolated cases or small series have been reported in the literature so far, the precise clinical spectrum of CNS-GvHD, its response to treatment and its prognosis are still poorly characterized, making its diagnosis and management particularly challenging. Furthermore, despite the growing evidence supporting the existence of acute CNS-GvHD, there is still no definition and diagnostic criteria for this entity.^7,8^ Since neurological complications have been shown to significantly increase morbidity and mortality after allo-HSCT,^9^ improving our understanding of CNS-GvHD is greatly needed.^10,11^ Here, we report the medical history, the clinical, biological and radiological findings, and the clinical course of the first large cohort of 66 patients diagnosed with possible CNS-GvHD (pCNS-GvHD).

## Materials and methods

### Study design and participants

In this retrospective study, we identified patients with CNS disorders for which the mechanism is thought to be immune-mediated, referred to as pCNS-GvHD, selected by predetermined diagnostic criteria. In this analysis, the term possible GvHD (or ‘atypical GvHD’) is consistent with the 2020 National Institutes of Health Consensus Project Task Force terminology to describe post-allo-HSCT immune-mediated manifestations of uncertain mechanism broadly.^10^ Both published and unpublished cases were solicited from authors who published in the field of GvHD and their networks and through the Société Francophone de Greffe de Moelle et Thérapie Cellulaire (SFGM-TC). Patients aged over 18 years with a history of allo-HSCT were included if they had presented clinical manifestations compatible with a CNS disorder, associated with at least two supportive criteria, and after reasonable exclusion of the alternative diagnoses (Table 1). Contrary to the 2010 consensus criteria,^6^ criteria used for this study were established to allow the inclusion of both patients with acute CNS-GvHD and those with chronic CNS-GvHD. Moreover, damage to other organs caused by chronic GvHD was not considered a mandatory criterion.

**Table 1:** Patient selection

Supportive criteria (at least two are needed for inclusion)	Exclusion criteria
Brain or spinal cord lesions visible on MRI at a neuroanatomical site compatible with the symptomatology	Differential diagnosis deemed more probable to explain the clinical observations, including: CNS infections CNS infiltration by neoplastic lesions Toxic, endocrine, metabolic, or deficiency-associated CNS disorders Stroke or intracranial haemorrhage without radiological or histopathological evidence of vasculitis Peripheral nervous system disorder responsible for the whole clinical picture Neurological disease already present before allo-HSCT and potentially responsible for the whole symptomatology
CSF WBC count >5 cells/mm^3^ or protein level >0.45 g/l	
Concomitant (within 30 days before or after) acute or chronic extra-neurological GvHD flare	
Clinical response to immunosuppressive therapy	
Parenchymal, perivascular or vascular mural lymphocyte infiltrates on histopathology	

To be eligible, patients must have presented signs or symptoms compatible with a CNS disorder after the age of 18 years, associated with at least two supportive criteria and no exclusion criteria. Allo-HSCT = allogeneic haematopoietic stem cell transplantation; GvHD = graft-versus-host disease; WBC = white blood cell.

This study was approved by the institutional review board of the University Hospital of Liège, Belgium (reference: 2022/246) and the SFGM-TC scientific council.

### Procedures

A case report form (CRF) was completed by the local investigator for each patient from centres non-affiliated with the SFGM-TC. For patients from centres affiliated with the SFGM-TC, available data were extracted from the SFGM-TC database, and additional data were collected through an adapted version of the CRF. Collected data comprised demographics, prior neurological, haematological and auto-immune disorders, data related to the haematologic disease and its treatments, allo-HSCT procedures, extra-CNS acute and chronic GvHD, clinical, biological, radiological and histopathological characteristics of the CNS disorder, as well as the clinical course of the disease, immunosuppressive treatments, response to treatments and clinical follow-up at 1 year. The neurological presentations were categorized into the following syndromes: meningitis, limbic encephalitis, extra-limbic encephalitis, brainstem encephalitis, myelitis, encephalomyelitis, multifocal demyelinating disease with neurologic deficits and CNS angiitis. Definitions used for these syndromes can be found in Appendix 1.^12-14^ Clinical response of pCNS-GvHD to immunosuppressive treatments was defined as clinical improvement or stabilization of a previously progressing disease. Relapse was defined as a recurrence of previous neurological signs or symptoms or the development of new signs or symptoms with the exclusion of alternative diagnoses. The degree of disability 1  year after pCNS-GvHD onset was categorized using the modified Rankin Disability Scale (mRS).^15,16^ The main cause of death was recorded based on the judgement of the local investigator and assigned to one of these five categories: relapse or progression of the underlying haematological disease; pCNS-GvHD; non-CNS GvHD; opportunistic infection; or other (to be specified).

### Objectives

The primary objective of the study was to describe the clinical, biological, radiological and histopathological presentation of pCNS-GvHD. Data were then further contrasted depending on whether pCNS-GvHD onset occurred before or after Day 100 following allo-HSCT or donor lymphocyte infusion (DLI). Secondary objectives included a description of the treatments and the resulting response, factors associated with response to treatment, 1-year overall survival (OS) after pCNS-GvHD onset, specific cause of death, factors associated with OS and with specific causes of death, and neurological sequelae.

### Statistical analyses

Categorical variables are reported as counts and percentages, and continuous variables as medians with interquartile range (IQ). Comparisons between subgroups were performed using Fischer’s exact test. Kaplan–Meier curves were used to describe survival. Univariate and stepwise Cox models were applied to find predictors of death during the year following pCNS-GvHD onset; the hazard ratio and associated 95% confidence interval [HR (95%CI)] are presented. Full details on Cox models are presented in Appendix 1. Cumulative events for specific causes of death were summarized using survival analyses with competing risks. Univariate and multivariate logistic binary regressions with stepwise selection of variables were performed to model the response to treatment depending on different selected factors. The odds ratio (OR) and 95%CI are displayed. The maximum available data was used in calculations, and no imputation of missing data was performed. All tests were two-sided and considered significant at an α-level of 0.05. Statistical analyses were conducted using Prism 10 (https://www.graphpad.com), SAS for Windows (version 9.4) and R (version 4.2.0).

## Results

### Patients

**Figure 1:**
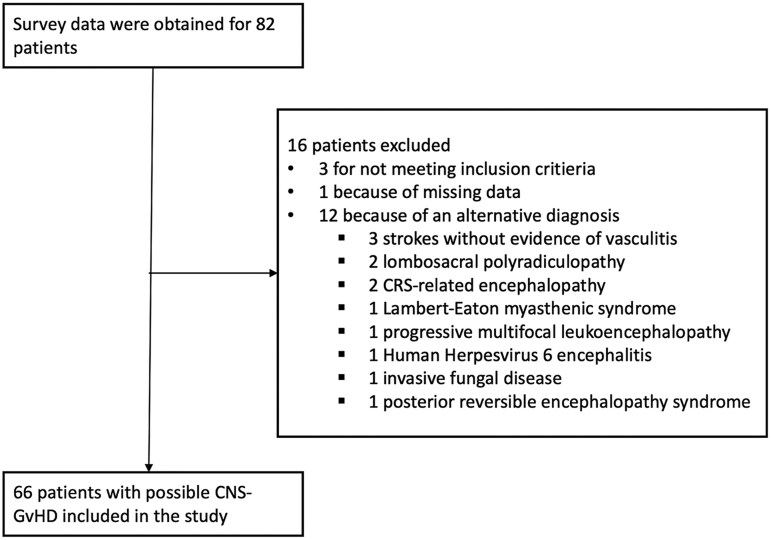
**Flow chart describing patients’ inclusion**. Survey data were received for a total of 82 patients. After revision of each case report form by the principal investigator, 16 patients were excluded: three patients because they did not meet inclusion criteria (less than two supportive criteria); one patient because of multiple missing data; and 12 patients because an alternate diagnosis was deemed more probable (three patients with stroke without radiological or histopathological evidence of vasculitis, two patients with lumbosacral polyradiculopathy, two patients with cytokine release syndrome (CRS)-associated encephalopathy, one patient with Lambert-Eaton myasthenic syndrome, one patient with progressive multifocal leukoencephalopathy, one patient with Human Herpesvirus 6 encephalitis, one patient with invasive fungal disease with brain involvement and one patient with posterior reversible encephalopathy syndrome). Sixty-six patients were therefore included. GvHD = graft-versus-host disease.

Data were received for a total of 82 patients. Among those, 16 patients were excluded: three because they did not meet the inclusion criteria; 12 because an alternative diagnosis was deemed more probable; and one because of missing data (Fig. 1). Hence, 66 patients from 14 countries presenting with pCNS-GvHD between 10 July 2006 and 30 June 2023 were included in the final analysis (Supplementary Fig. 1). Among them, nine cases had previously been published in the literature.^17-21^ Patients and transplant-related characteristics are displayed in Table 2. Sex at birth was male for 43 patients (65%) and median age at pCNS-GvHD onset was 57 years (IQ_25–75_ 42–65). Most patients (92%) were transplanted for haematological malignancies. The conditioning regimen was intended to be myeloablative for 22 patients (33%) and 14 patients (21%) received total body irradiation as part of the regimen. The transplant consisted of mobilized peripheral blood stem cells for most patients [53 patients (80%)], and the donor was unrelated in most cases [44 patients (67%), HLA-matched in 34 cases and HLA-mismatched in 10]. For three patients (5%), it was the second allo-HSCT. Three patients (5%) received donor lymphocyte infusions (DLIs) after the transplantation.

**Table 2:** Patient and transplant-related characteristics

Patients and transplant characteristics	All cases (*N* = 66)	pCNS-GvHD ≤100 days (*N* = 27)	pCNS-GvHD >100 days (*N* = 39)
Male sex, *n* (%)	43 (65%)	16 (59%)	27 (69%)
Age at pCNS-GvHD (years), median (IQ_25–75_)	57 (42–65)	62 (50–67)	56 (42–64)
Underlying disease^a^, *n* (%)			
Myeloid malignancies	47 (71%)	24 (89%)	23 (59%)
Lymphoid malignancies	14 (21%)	2 (7%)	12 (31%)
Non-malignant diseases	5 (8%)	1 (4%)	4 (10%)
CNS disorder prior allo-HSCT^b^, *n* (%)	8 (12%)	3 (11%)	5 (13%)
Immune-mediated disorder prior allo-HSCT^c^, *n* (%)	6 (9%)	2 (7%)	4 (10%)
Conditioning regimen before allo-HSCT	–	–	–
Myeloablative, *n* (%)	22 (33%)	4 (15%)	18 (46%)
TBI-based, *n* (%)	14 (21%)	3 (11%)	11 (28%)
Source of stem cells, *n* (%)	–	–	–
Mobilized peripheral blood stem cells	53 (80%)	24 (89%)	29 (74%)
Bone marrow	11 (17%)	2 (7%)	9 (23%)
Cord blood	2 (3%)	1 (4%)	1 (3%)
Donor type, *n* (%)	–	–	–
Related, HLA-matched	11 (17%)	3 (11%)	8 (21%)
Related, HLA-haploidentical	11 (17%)	4 (15%)	7 (18%)
Unrelated, HLA-matched	34 (52%)	16 (59%)	19 (49%)
Unrelated, HLA-mismatched	10 (15%)	4 (15%)	4 (10%)
Donor-recipient sex mismatch (female for male), *n* (%)	23 (35%)	6 (22%)	17 (44%)
CMV reactivation after allo-HSCT, *n* (%)	19 (37%^d^)	8 (38%^d^)	11 (37%^d^)
EBV reactivation after allo-HSCT, *n* (%)	12 (24%^d^)	3 (14%^d^)	9 (30%^d^)
Complete donor chimerism at last bone marrow aspiration before pCNS-GvHD onset, *n* (%)	42 (84%^e^)	16 (76%^e^)	26 (90%^e^)
DLI before pCNS-GvHD, *n* (%)	3 (5%)	3 (11%)	0 (0%)
Delay between allo-HSCT/DLI and pCNS-GvHD (days), median (IQ_25–75_)	149 (48–321)	40 (14–70)	279 (154–448)

allo-HSCT = allogeneic haematopoietic stem cell transplantation; aGvHD = acute graft-versus-host disease; cGvHD = chronic graft-versus-host disease; CMV = cytomegalovirus; DLI = donor lymphocyte infusion; EBV = Epstein-Barr virus; IQ = interquartile range; pCNS-GvHD = possible CNS graft-versus-host disease.

^a^Underlying disease: acute myeloblastic leukaemia (25 patients), myeloproliferative neoplasm (17 patients), acute lymphoblastic leukaemia (seven patients), myelodysplastic syndrome (five patients), non-Hodgkin’s lymphoma (three patients), inherited bone marrow failure (two patients), Hodgkin’s lymphoma (two patients), primary immune deficiency (two patients), multiple myeloma (two patients) and aplastic anaemia (one patient).

^b^History of CNS disorder prior to allo-HSCT: stroke (two patients), chemotherapy-induced toxic encephalopathy (two patients), essential tremor (one patient), epilepsy following the cure of an aneurysm of the right middle cerebral artery (one patient), subarachnoid haemorrhage (one patient) and traumatic acute subdural haematoma (one patient).

^c^History of non-haematological immune-mediated disorder prior to allo-HSCT: psoriasis, pulmonary alveolar proteinosis, auto-immune uveitis, erythema nodosum, rheumatoid arthritis and ulcerative colitis (one patient each).

^d^Data on CMV and EBV reactivations were available for 51 patients (21 with pCNS-GvHD before and 30 with pCNS-GvHD after Day 100).

^e^Data on donor chimerism were available for 50 patients (21 with pCNS-GvHD before and 29 with pCNS-GvHD after Day 100).

Non-CNS acute GvHD occurred in 50 patients (76%), 24 of them diagnosed within 1 month before or after pCNS-GvHD onset. Prior or active chronic GvHD was present in 27 patients (41%), 20 of them diagnosed within 1 month from pCNS-GVHD onset. The main characteristics related to extra-CNS GvHD are displayed in Supplementary Table 1. The median time between allo-HSCT or DLI and pCNS-GvHD onset was 149 days (IQ_25–75_ 48–321). It occurred before Day 100 following allo-HSCT or DLI in 27 patients (41%) and after Day 100 in 39 patients (59%).

### Clinical characteristics of possible CNS-graft-versus-host disease

Neurological manifestations at initial presentation are listed in Table 3; most frequent were cognitive and/or behavioural impairment (27 patients, 41%), paresis of one or more limb(s) (14 patients, 21%), altered consciousness (13 patients, 20%), sensory impairment (12 patients, 18%) and headache (10 patients, 15%). CNS manifestations that occurred at any time during the disease are displayed in Supplementary Table 2. Cognitive and/or behavioural impairments were the most frequent clinical findings (48 patients, 73%). Multiple clinical neurological manifestations were present in 31 patients (47%) at initial presentation and occurred in most patients (64 patients, 97%) during the course of the disease. Clinical presentation did not differ notably between patients with pCNS-GvHD occurring before or after Day 100 following transplantation or DLI. Of note, concomitant peripheral nervous system manifestations of chronic GvHD, as defined by the Consensus Conference on Clinical Practice in chronic GvHD,^4^ occurred in nine patients (14%) (Supplementary Table 1).

**Table 3:** Clinical manifestations of possible CNS graft-versus-host disease at initial presentation and neurological sequelae among surviving patients 1 year after onset

Clinical signs/symptoms	All cases	pCNS-GvHD ≤100 days	pCNS-GvHD >100 days
**At initial presentation**	***N* = 66**	***N* = 27**	***N* = 39**
Cognitive and/or behavioural impairment, *n* (%)	27 (41%)	14 (52%)	13 (33%)
Speech impairment, *n* (%)	5 (8%)	2 (7%)	3 (8%)
Motor impairment, *n* (%)	14 (21%)	4 (15%)	10 (26%)
One or both upper limb(s)	6 (9%)	2 (7%)	4 (10%)
One or both lower limb(s)	12 (18%)	3 (11%)	9 (23%)
Gait impairment, *n* (%)	9 (14%)	3 (11%)	6 (15%)
Vision impairment, *n* (%)	7 (11%)	1 (4%)	6 (15%)
Sensory impairment, *n* (%)	12 (18%)	2 (7%)	10 (26%)
Epileptic seizure, *n* (%)	2 (3%)	1 (4%)	1 (3%)
Headache, *n* (%)	10 (15%)	6 (22%)	4 (10%)
Hyperkinetic movement disorder, *n* (%)	8 (12%)	6 (22%)	2 (5%)
Cranial nerve disorder, *n* (%)	5 (8%)	2 (7%)	3 (8%)
Urinary or anal sphincter dysfunction, *n* (%)	3 (5%)	1 (4%)	2 (5%)
Disorder of consciousness, *n* (%)	13 (20%)	8 (30%)	5 (13%)
**Neurological sequelae 1 year after disease onset**	***N* = 23**	***N* = 5**	***N* = 18**
Cognitive and/or behavioural impairment, *n* (%)	8 (35%)	1 (20%)	7 (39%)
Speech impairment, *n* (%)	1 (4%)	0 (0%)	1 (6%)
Motor impairment, *n* (%)	3 (13%)	1 (20%)	2 (11%)
One or both upper limb(s)	1 (4%)	1 (20%)	0 (0%)
One or both lower limb(s)	2 (9%)	0 (0%)	2 (11%)
Gait impairment, *n* (%)	7 (30%)	0 (0%)	7 (39%)
Vision impairment, *n* (%)	1 (4%)	0 (0%)	1 (6%)
Sensory impairment, *n* (%)	2 (9%)	0 (0%)	2 (11%)
Epileptic seizure, *n* (%)	2 (9%)	0 (0%)	2 (11%)
Headache, *n* (%)	2 (9%)	0 (0%)	2 (11%)
Hyperkinetic movement disorder, *n* (%)	1 (4%)	0 (0%)	1 (6%)
Cranial nerve disorder, *n* (%)	1 (4%)	0 (0%)	1 (6%)
Urinary or anal sphincter dysfunction, *n* (%)	3 (13%)	0 (0%)	3 (17%)
Disorder of consciousness, *n* (%)	1 (4%)	0 (0%)	1 (6%)
No clinical neurological sequelae, *n* (%)	7 (30%)	3 (60%)	6 (33%)

As multiple clinical manifestations or sequelae may be present, numbers may not sum to group totals, or percentages add to 100%. pCNS-GvHD = possible CNS graft-versus-host disease.

### Radiological characteristics

**Figure 2:**
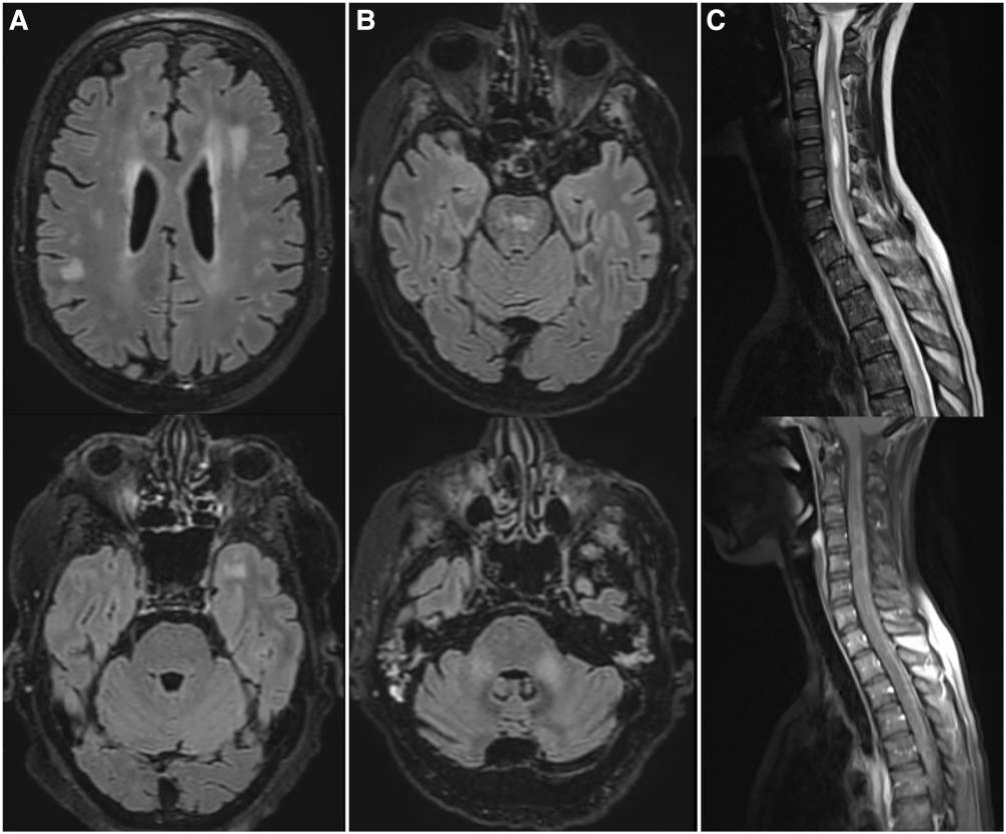
**Clinical course and MRI findings of three illustrative cases**. (**A**) Patient 1 was admitted for behavioural changes and cognitive decline associated with headache and blurred vision progressing over weeks. Two years earlier, he had received an allogeneic haematopoietic stem cell transplantation (allo-HSCT) for primary myelofibrosis. Those symptoms were concomitant with the development of classic signs of mouth and skin chronic graft-versus-host disease (cGvHD). Brain MRI showed multifocal T2/fluid-attenuated inversion recovery (FLAIR)-hyperintense white matter lesions. CSF was unremarkable. After ruling out infectious differential diagnoses, including notably progressive multifocal leukoencephalopathy, the patient was treated with a combination of high-dose corticosteroids, rituximab and cyclophosphamide, which allowed complete resolution of the symptomatology. (**B**) Patient 2 was admitted to the intensive care unit for a decreased level of consciousness and movement disorders 2 weeks following allo-HSCT for myelodysplastic syndrome. Brain MRI showed T2/FLAIR hyperintense lesions involving the pons and the cerebellar peduncles, with areas of restricted diffusion. CSF analysis revealed increased white blood cell (WBC) count (65 cells/mm^3^) and high protein level (1.355 g/l). There was no sign of extra-neurological GvHD. The patient was treated with weekly intrathecal infusions of corticosteroids associated with systemic mycophenolate, which allowed improvement of the symptomatology and complete disappearance of the brain lesions. (**C**) Patient 3 presented with tetraparesis, proprioceptive ataxia and sphincter dysfunction progressing over days. Two years earlier, she had been treated with allo-HSCT for acute lymphoblastic leukaemia. She had no previous or active extra-neurological GvHD. Spinal cord MRI showed a longitudinally extensive T2-weighted hyperintense lesion extending from level C1 to the conus medullaris (*top*), with areas of enhancement after gadolinium injection (*bottom*). Brain MRI was normal. CSF analyses showed increased WBC count (17 cells/mm^3^) and protein level (2.564 g/l). She was treated with high-dose systemic corticosteroids and tacrolimus, which allowed complete resolution of the clinical symptoms and regression of the lesions visualized with MRI.

Brain MRI was performed in 65 cases (98%), and lesions were found at a neuroanatomical site compatible with the symptomatology in 37 (57%) of them (Table 4 and Fig. 2). Intraparenchymal lesions were found in 35 patients and extra-parenchymal lesions in three, including leptomeningitis for two patients and pachymeningitis for one. Among the 35 patients with intraparenchymal brain lesions, 29 (83%) had multiple lesions, while six patients had a single lesion. Supratentorial lesions were present in 30 patients and infratentorial lesions in 15. Nineteen of thirty-five patients (54%) presented non-confluent white matter lesions, 14 (40%) confluent white matter lesions, one (3%) a pseudo-tumoural lesion and one (3%) an acute ischemic lesion. Contrast enhancement after gadolinium injection of at least one lesion was found in 12 patients (34%). Brain CT was performed in 44 patients but abnormalities compatible with the symptomatology were found only in three of them (7%).

**Table 4:** Biological and radiological characteristics of the whole cohort and of subgroups depending on the delay between allogeneic haematopoietic stem cell transplantation or donor lymphocyte infusion and CNS- graft-versus-host disease onset

MRI and CSF characteristics	All patients	Patients with CNS-GvHD onset before Day 100	Patients with CNS-GvHD onset after Day 100
**Patients with brain MRI results available, *N***	**65**	**26**	**39**
Brain lesions seen with MRI compatible with symptomatology, *n* (%) Among these:	35 (54%)	11 (42%)	24 (62%)
Supratentorial lesions, *n* (%)	30 (86%)	7 (64%)	23 (96%)
Infratentorial lesions, *n* (%)	15 (43%)	6 (54.5%)	9 (38%)
Contrast-enhancing lesions, *n* (%)	12 (34%)	2 (7.7%)	10 (42%)
Multiple lesions, *n* (%)	29 (83%)	6 (55%)	23 (96%)
Type of lesions, *n* (%)	–	–	–
Separate oval or punctuate white matter lesions	19 (54%)	5 (45%)	14 (58%)
Confluent white matter lesions	14 (40%)	6 (55%)	8 (33%)
Acute ischemic lesions	1 (3%)	0	1 (4%)
Pseudo-tumoral lesions	1 (3%)	0	1 (4%)
Extra-parenchymal intracranial lesions, *n* (%)	3 (5%)	1 (4%)	2 (5%)
**Patients with spinal cord MRI results available, *N***	**36**	**11**	**25**
Spinal cord lesions seen with MRI, *n* (%) Among these:	7 (19%)	0 (0%)	7 (28%)
Longitudinally extensive, *n* (%)	6 (86%)	0 (0%)	6 (86%)
Contrast-enhancing lesions, *n* (%)	6 (86%)	0 (0%)	6 (86%)
Multiple lesions, *n* (%)	5 (71%)	0 (0%)	5 (71%)
**Patients with brain CT results available, *N***	**44**	**19**	**25**
Brain lesions seen with CT compatible with symptomatology, *n* (%)	3 (7%)	0 (0%)	3 (12%)
**Patients with CSF results available, *N***	**64**	**25**	**39**
CSF WBC count >5/mm^3^, *n* (%)	36 (56%)	15 (60%)	21 (54%)
CSF WBC count 6–20/mm^3^, *n* (%)	20 (31%)	6 (24%)	14 (36%)
CSF WBC count 21–50/mm^3^, *n* (%)	11 (17%)	5 (20%)	6 (15%)
CSF WBC count 51–200/mm^3^, *n* (%)	4 (6%)	4 (16%)	0 (0%)
CSF WBC count >200/mm^3^, *n* (%)	1 (2%)	0 (0%)	1 (3%)
Among these, WBC count, cells/mm^3^, median (IQ_25–75_)	18 (10–43.25)	30 (12–60)	14 (7–40)
CSF protein level >0.45 g/l, *n* (%)	51 (80%)	19 (76%)	32 (82%)
CSF protein level, g/l, median (IQ_25–75_)	0.79 (0.51–1.31)	0.6 (0.45–1.32)	0.9 (0.65–1.31)
CSF glucose level <0.45 mg/dl, *n* (%)	6 (11%^a^)	1 (5%^a^)	5 (14%^a^)
CSF glucose level, mg/dl, median (IQ_25–75_)	60.5 (54–75)	70 (55–83)	59 (53.5–66)
CSF oligoclonal bands, *n* (%)	16 (47%^b^)	3 (30%^b^)	13 (54%^b^)

IQ = interquartile range; pCNS-GvHD = possible CNS graft-versus-host disease; WBC = white blood cell.

^a^CSF glucose level was available for 56 patients (20 with pCNS-GvHD before and 36 with pCNS-GvHD after Day 100).

^b^Data on CSF oligoclonal bands were available for 34 patients (10 with pCNS-GvHD before and 24 with pCNS-GvHD after Day 100).

Spinal cord MRI was performed in 36 patients (11 with pCNS-GvHD onset before Day 100 following transplantation or DLI and 25 with pCNS-GvHD onset after Day 100) and showed abnormalities in seven (19%) of them. Five patients had multiple spinal cord lesions, while two had a single lesion. Most lesions (six of seven patients) were longitudinally extensive, defined as lesions extending over three or more vertebrae. Most lesions (six of seven patients) showed enhancement after gadolinium injection.

More patients with visible lesions on MRI exhibited pCNS-GvHD after Day 100 than before Day 100 following transplantation [29 of 39 (74%) patients and 11 of 26 patients (42%), respectively, *P* = 0.018]. Interestingly, spinal cord lesions were observed exclusively among patients who exhibited pCNS-GvHD >100 days after allo-HSCT or DLI. Additionally, more patients presented with multiple brain lesions in the group [23 of 24 (96%) versus 6 of 11 (55%) patients] exhibiting pCNS-GvHD before Day 100, *P* = 0.0071.

### CSF characteristics

CSF was sampled and analysed in 64 patients (Table 4). Thirty-six (56%) showed an increased white blood cell (WBC) count (>5 cells/μl), with a median of 18 cells/μl (IQ_25–75_ 10–43.25). Regarding the nature of WBC in the CSF, most patients had a predominantly lymphocytic profile (>90% of WBC). Nine patients had a predominantly lymphocytic profile, though more mixed, with over 50% lymphocytes and the remainder consisting of neutrophils and monocytes. Additionally, two patients had a predominantly lymphocytic profile associated with eosinophils (accounting for 18% and 5% of the WBC in the CSF, respectively). Finally, one patient had a profile primarily composed of neutrophils. The median CSF protein level was 0.79 g/l (IQ_25–75_ 0.51–1.31), with 51 patients (80%) showing a protein level over 0.45 g/l. A decreased CSF glucose level (under 45 mg/dl) was infrequent (11% of patients). Among the 34 patients for whom the information was available, 16 (47%) had oligoclonal bands in the CSF. Antibodies directed against glial acidic fibrillary protein (GFAP) were found in the CSF of one patient, while antibodies directed against leucine-rich, glioma-inactivated 1 (LGI1) and contactin-associated protein-like 2 (CASPR2) were found in the sera of two and one patient(s), respectively. CSF analyses were similar between patients exhibiting pCNS-GvHD before and after Day 100 following allo-HSCT or DLI. Altogether, 57 patients (86%), including 19 (70%) with pCNS-GvHD that occurred before and 37 (95%) with pCNS-GvHD that occurred after Day 100, had abnormal MRI and/or increased CSF WBC count.

### Histopathological analyses

Histopathological analyses were performed on 12 specimens from 11 patients. Biopsy specimens were obtained from eight patients (seven from brain lesions and one from a spinal cord lesion), and autopsy specimens were obtained from four patients, including one patient with both biopsy and autopsy specimens available. Histopathological analyses were considered suggestive of CNS-GvHD by the local pathologist for 10 specimens. Among the two analyses considered non-suggestive, one was performed on an autopsy specimen from a patient who had been treated with several lines of immunosuppressive therapy and was in complete clinical remission of the CNS disorder at the time of death. The other non-suggestive analysis was performed on a brain biopsy and only showed reactional gliosis, but the subsequent autopsy showed evidence of encephalitis. Apart from these two, all brain specimens showed parenchymal and perivascular infiltration by CD3+ cells (T cells) and CD163+ cells (macrophages). Infiltration by CD8+ T cells (cytotoxic T cells) was predominantly reported, while CD4+ T cells (helper T cells) were present but rare. CD20+ cells (B cells) were exceptionally observed. All specimens showed reactive gliosis, and necrosis was observed in four. Lympho-histiocytic infiltration of the walls of arterioles and capillaries was observed in three patients, with the presence of fibrinoid necrosis of the vessels in two of them. In addition, the spinal cord specimen also showed extensive demyelination.

### Treatment

Immunosuppressive therapy was administrated to 64 patients (97%). Drugs prescribed as part of the first-line regimen (described in Table 5) comprised corticosteroids for most (58 of 64 treated patients (91%)]. Twenty-six patients (41%) received at least two treatments concomitantly as part of the initial therapeutic regimen. A clinical response to this first-line therapy was observed in 51 patients (80%), among which 17 (27%) showed complete clinical recovery, 30 (47%) experienced partial improvement and four (6%) showed stabilization of the previously progressing disease. On univariate logistic regression model, the probability of response to treatment was significantly lower among patients with altered consciousness [OR (95%CI): 0.16 (0.04–0.62); *P* = 0.008] and patients with multiple clinical findings at initial presentation [OR (95%CI): 0.21 (0.05–0.86); *P* = 0.03]. In the multivariate model, only disorder of consciousness at initial presentation was associated with a reduced probability of response to treatment [OR (95%CI): 0.10 (0.02–0.47); *P* = 0.004] (Supplementary Table 3). Clinical relapse was observed in 16 patients (30% of responders), usually during the treatment taper or in the following 3 months. CSF was sampled after treatment for 23 patients, among which six samples (26%) showed complete resolution of the previously observed abnormalities, nine (39%) partial amelioration (defined as 50% reduction of the WBC count and/or protein level) and eight (35%) showed no improvement. MRI was repeated for 27 patients, among whom one (4%) showed complete disappearance of the lesions, 10 (37%) reduction in the size or number of lesions, 12 (44%) stability and four (15%) progression of the lesions. Additional lines of treatment were administrated to 23 patients, either for a relapse (14 patients) or for a response to first-line therapy judged insufficient (nine patients).

**Table 5:** Treatments administrated as first-line regimen

First-line therapy	All cases	pCNS-GvHD ≤100 days	pCNS-GvHD >100 days
Total number of treated patients, *N*	64	26	38
Corticosteroids, *n* (%)	58 (91%)	23 (88%)	35 (92%)
Methylprednisolone 500 to 1000 mg/day^a^	23	8	15
Methylprednisolone 1 to 2 mg/kg/day^a^	22	12	10
Prednisone 1 mg/kg/day^a^	7	0	7
Other corticosteroids regimen^b^	6	3	3
Calcineurin inhibitor, *n* (%)	7 (11%)	4 (15%)	3 (8%)
Mycophenolate mofetil, *n* (%)	6 (9%)	3 (12%)	3 (8%)
Intravenous immunoglobulins, *n* (%)	8 (13%)	1 (4%)	7 (18%)
Plasma exchanges, *n* (%)	6 (9%)	1 (4%)	5 (13%)
Rituximab, *n* (%)	5 (8%)	1 (4%)	4 (11%)
Cyclophosphamide, *n* (%)	4 (6%)	2 (8%)	2 (5%)
Ruxolitinib, *n* (%)	4 (6%)	1 (4%)	3 (8%)
Tocilizumab, *n* (%)	1 (2%)	1 (4%)	0 (0%)
Fingolimod, *n* (%)	1 (2%)	0 (0%)	1 (3%)
Sirolimus, *n* (%)	1 (2%)	0 (0%)	1 (3%)
Combination of at least two treatments, *n* (%)	26 (41%)	8 (31%)	18 (47%)

pCNS-GvHD = possible CNS graft-versus-host disease. As multiple treatments may be administrated, numbers may not sum to group totals or percentages add to 100%. pCNS-GvHD possible CNS involvement in acute and chronic graft-versus-host disease.

^a^Initial dose.

^b^Other regimens include methylprednisolone 40 mg given intrathecally weekly (two patients), methylprednisolone 0.5 mg/kg/day (one patient), prednisone 0.5 mg/kg/day (one patient), dexamethasone 20 mg/day (one patient), dexamethasone 40 mg/day (one patient).

### Outcome

**Figure 3:**
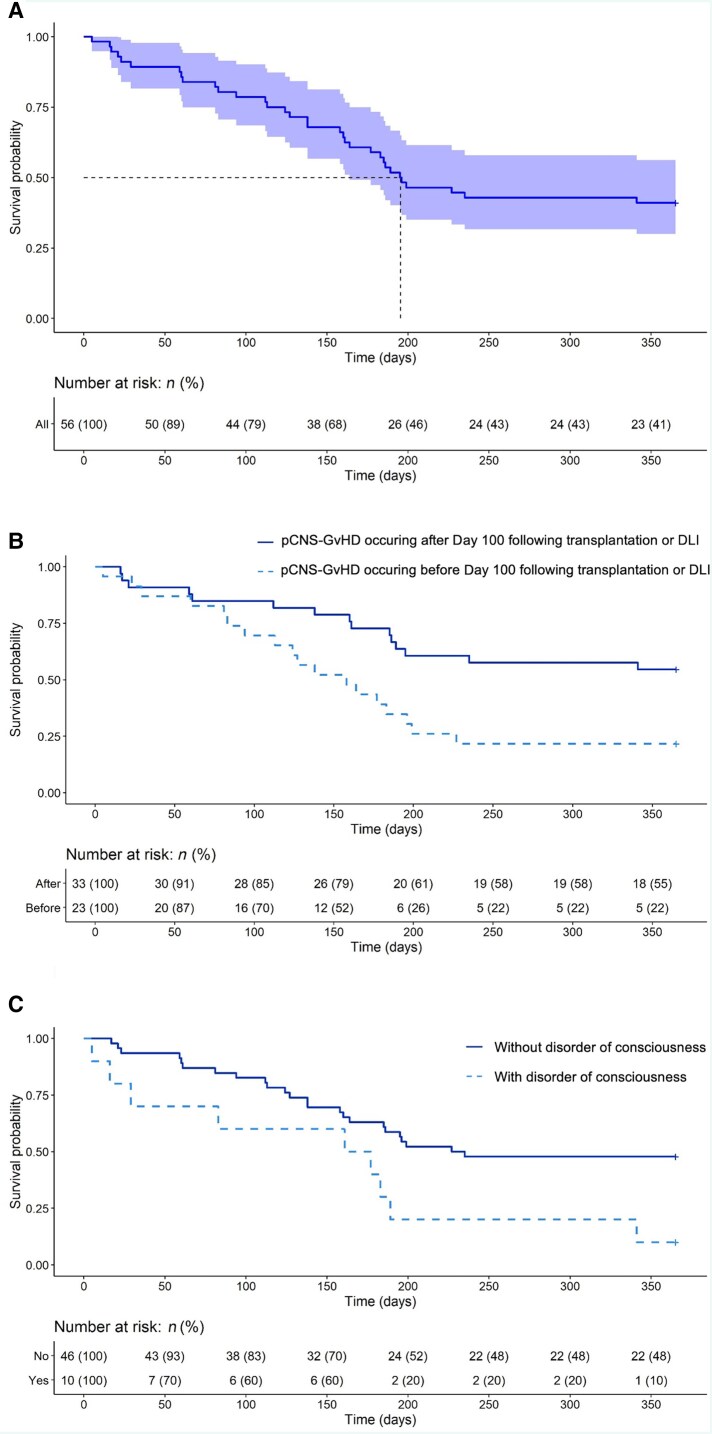
**One-year probability of survival following possible CNS-graft-versus-host disease onset.** (**A**) Whole cohort (light blue area indicates 95% confidence interval), (**B**) according to the interval between allogeneic haematopoietic stem cell transplantation (allo-HSCT) and possible CNS-graft-versus-host disease (pCNS-GvHD) onset and (**C**) according to the presence or not of altered consciousness at initial presentation.

One-year follow-up was available for 56 patients. One-year OS following pCNS-GvHD onset was 41% (23 patients), with a median OS of 196 (95%CI: 164–NE) days. On both univariate and multivariate Cox models, 1-year OS probability was significantly lower among patients with disorder of consciousness at initial presentation [HR (95%CI): 2.5 (1.2–5.4); *P* = 0.019 for univariate analyses, and 3.0 (1.3–6.7); *P* = 0.0077 for multivariate analyses] and those with pCNS-GvHD occurring before Day 100 [2.5 (1.2–4.9); *P* = 0.01, and 2.1 (1.0–4.5; *P* = 0.041 for univariate and multivariate analyses, respectively] (Supplementary Table 4). The probability of survival during the first year following pCNS-GvHD onset is shown in Fig. 3A for the whole cohort and Fig. 3B and C based on the time since allo-HSCT or DLI, and the presence of altered consciousness at presentation, respectively. Of note, the two patients who received no treatment both died of pCNS-GvHD 17 and 83 days after symptom onset.

The cause of death was attributed to pCNS-GvHD in 15 of 33 patients (47%), opportunistic infections in 13 patients (41%) and progression of the underlying disease and extra-neurological GvHD in one patient (3%) each (Supplementary Fig. 2). In addition, one patient died of diffuse alveolar haemorrhage of unknown aetiology, one of cardiac arrhythmia and one was found dead at home, with no known aetiology. While none of the factors considered was significantly associated with the probability of death specifically due to pCNS-GvHD (Supplementary Table 5), pCNS-GvHD occurring before day 100 following allo-HSCT or DLI was associated with an increased probability of death due to opportunistic infections [HR (95%CI): 3.83 (1.20–12.21); *P* = 0.02] (Supplementary Table 6).

Among surviving patients, 14 (61%) had neurological sequelae (Table 3), most frequently cognitive and behavioural sequelae and walking impairment. Nine patients (39% of surviving patients) had a modified Rankin Scale score of 0–1, eight (35%) had a score of 2, three (13%) had a score of 3 and one (4%) had a score of 4.

### 2010 criteria for chronic CNS-GvHD

Twenty-seven patients (41%) had extra-CNS chronic GvHD at the time of inclusion and, therefore, met the 2010 criteria for the diagnosis of chronic pCNS-GvHD.^6^ The characteristics of these patients are provided in the online supplementary material (Supplementary Tables 7–10). There was no significant difference in clinical, biological, and radiological characteristics, nor in response to immunosuppressive therapy or 1-year OS between patients who met 2010 criteria and those with pCNS-GvHD occurring after Day 100 not meeting these criteria (Supplementary Tables 11 and 12). Of note, seven of the ten patients with pCNS-GvHD occurring after Day 100 without extra-CNS manifestations of chronic GvHD at that time developed it subsequently.

### Syndromic approach

Among the 66 patients with pCNS-GvHD, 42 (64%) presented with extra-limbic encephalitis, nine (14%) had multifocal demyelinating disease with neurologic deficits, five (8%) had encephalomyelitis, four (6%) had brainstem encephalitis, three (5%) had myelitis, two (3%) had meningitis and one (2%) had CNS angiitis. None of the patients presented with limbic encephalitis. Among the patients with extra-limbic encephalitis or encephalomyelitis, eight met the criteria for acute disseminated encephalomyelitis (ADEM) at initial presentation, with one of them progressing to a multiphasic form.^12,22^ There was no significant difference in response to immunosuppressive therapy or 1-year OS between the different syndromes (Supplementary Tables 13 and 14). Additionally, we did not find any statistically significant difference in the occurrence of syndromes between patients with pCNS-GvHD occurring before or after Day 100 following allo-HSCT or DLI (Supplementary Table 15), although it is worth noting that myelitis and CNS angiitis only occurred after Day 100 following transplantation.

It should be noted that patients who presented with antibodies generally associated with autoimmune encephalitis exhibited clinical and radiological characteristics similar to those observed with the same antibodies in the non-transplanted population. For instance, the patient with anti-GFAP antibodies presented with encephalopathy accompanied by movement disorders and hyperintensities in the basal ganglia, responding to corticosteroids while the patient with anti-LGI1 antibodies presented with encephalitis featuring focal seizures and responding to plasmapheresis and rituximab.^23-25^

## Discussion

Progress in our overall understanding of CNS-GvHD is slow because of its perceived rarity and the difficulty to make the diagnosis. Available criteria,^6^ established in 2010 and deriving from the criteria proposed 1 year earlier by Openshaw,^26^ only allow the ‘possible’ diagnosis of chronic CNS-GvHD in the presence of typical clinical signs of extra-neurological chronic GvHD. However, in several situations reported in the literature,^17,20^ patients do not show typical signs of chronic GVHD, and yet the treating physician estimates that CNS-GvHD is the most probable diagnosis, which might reflect a certain lack of sensitivity of these criteria in clinical practice.^10^ It is important to note that the decision to make the presence of extra-neurological chronic GvHD involvement mandatory for diagnosing CNS-GvHD was arbitrary, based on expert consensus and did not rely on solid scientific data. In addition, the 2010 criteria do not permit the diagnosis of acute CNS-GvHD. Thus, we decided to use more permissive inclusion criteria for our study, allowing the diagnosis of acute and chronic pCNS-GvHD with or without extra-neurological involvement, not because we aimed to describe a new entity, but rather because the 2010 criteria seem insufficiently sensitive for clinical practice. In consequence, more than half of our patients did not meet the 2010 criteria. The main risk of adopting more permissive criteria in clinical practice is to unduly treat patients without CNS-GvHD with immunosuppressive therapy and therefore unnecessarily expose them to potentially life-threatening adverse events. Reassuringly, neither response to immunosuppressive therapy nor 1-year survival significantly differed between patients who met the 2010 criteria and those who did not. Therefore, our criteria might have the double benefit of allowing the diagnosis of acute pCNS-GvHD and permitting the diagnosis of chronic pCNS-GvHD in more patients without increasing the proportion of patients unnecessarily exposed to immunosuppressive therapy. Further studies are needed to validate the benefit of these criteria in clinical practice.

The terminology for presumed immune-mediated CNS manifestations described in this report could be a matter of debate. GvHD is characterized by a failure of immune tolerance in a context of allo-reactivity.^27^ However, in addition to allo-reactivity, immune dysregulation observed following allo-HSCT can favour *de novo* auto-immunity, leading to diseases resembling those observed in non-transplanted patients, such as myasthenia gravis.^10,28^ In such situations, the direct role of allogeneic haematopoietic chimerism is unknown and qualifying them as part of GvHD is open to debate. The pathophysiology of the manifestations described here is unknown and might implicate both allo-immune and auto-immune mechanisms, as highlighted by the presence of antibodies usually associated with auto-immune encephalitis in four patients. In counterpart, allo-reactivity and auto-immunity are intrinsically linked and factors underlying classic auto-immune diseases, such as molecular mimicry or bystander activation related to the microbiome diversity, have been shown to play a major role in the pathophysiology of acute and chronic GvHD.^11,29,30^ In addition, post-alloHSCT auto-immune conditions usually occur alongside GvHD and some auto-immune diseases, such as systemic sclerosis, share high-level similarities with classical presentations of chronic GvHD.^6,31,32^ The report of the 2020 NIH Consensus Project Task Force decided to use the term ‘atypical GvHD’ for post-allo-HSCT immune-mediated manifestations of uncertain mechanism, a term we align with.^10^

Clinical, biological and radiological characteristics of pCNS-GvHD, as well as its response to treatment, were highly variable, in line with previous reports depicting multiple presentations of CNS-GvHD.^6,10^ This probably reflects that the entity described here is heterogeneous and might implicate multiple pathophysiological mechanisms. Because there is no robust objective biomarker for CNS-GvHD,^26^ our diagnosis relied on the accumulation of supportive criteria after exclusion of alternative diagnoses. Hence, we cannot irrevocably exclude that we may have included in our analysis some patients with disorders other than genuine CNS-GvHD, such as atypical drug-related toxicities which can take many aspects and trigger inflammation. On the other hand, as discussed above, application of our criteria did not increase the proportion of patients unduly exposed to immunosuppressive therapy compared to previously proposed criteria. Nevertheless, because the relation between brain dysfunction and genuine GvHD still needs to be established, we used the term ‘possible CNS-GvHD’. Further studies aiming to identify objective and robust markers of the disease that would allow us to make the definitive diagnosis of CNS-GvHD are highly needed.

Occurrence of pCNS-GvHD before Day 100 following allo-HSCT was associated with a reduced 1-year survival. However, overall and non-relapse mortality following allo-HSCT is already higher in the early post-engraftment period.^33^ Occurrence of the disease before Day 100 was also associated with an increased risk of death specifically due to opportunistic infections but not to mortality due to pCNS-GvHD itself. Hence, the increased mortality of patients with pCNS-GvHD occurring early after transplantation seems not to be directly due to a more aggressive form of disease but rather to a state of greater vulnerability to opportunistic infections, intrinsic to the early engraftment period. The presence of a disorder of consciousness at presentation was also associated with an increased mortality. However, whether this presentation reflects an aggressive disease or a late presentation is uncertain. Prospective trials will be needed to assess factors truly associated with more aggressive pCNS-GvHD.

We acknowledge that our study has several limitations intrinsic to its observational, retrospective design. The study was multicentric and international, which improves generalizability of the findings although data obtained at each site was heterogenous and limited statistical analyses. The non-comparative design of the study did not allow us to assess the incidence of the disease neither factors associated with its occurrence. Also, the design of the study, which relied on a call for cases among specialized centers and not a systematic review of their database, may have resulted in a selection bias. In counterpart, this is a unique study including a large cohort of patients and allowing, compared to previous small series or review of the literature,^17,34^ a more accurate description of CNS-GvHD, based on a standardized CRF. It is also the first study comprising systematic collection of follow-up data 1  year after CNS-GvHD onset, allowing the description of the prognosis of the disease as well as factors associated with a poor outcome. Noteworthy, our study is the first to compare acute and chronic CNS-GvHD, notably demonstrating distinct radiological presentations and prognosis.

In conclusion, this study supports that immune-mediated CNS manifestations may occur following allo-HSCT. These can be associated with both acute and chronic GvHD. The clinical spectrum at initial presentation is highly variable, as are its radiological and biological characteristics. The prognosis is grim, with a 1-year survival of 41% and neurological sequelae in 61% of surviving patients.

## Supplementary Material

awae340_Supplementary_Data

## Data Availability

Data requests should be sent to Nicolas Lambert. Data access must be approved by the Belgian data protection authority. For more information, see https://www.dataprotectionauthority.be/citizen

## Appendix 1

**CNS-GVHD Study Group collaborators**

Further details are provided in the Supplementary material.

Nicolas Lambert, Florence Forte, Majdouline El Moussaoui, Justine Monseur, Nicole Raus, Alexey Polushin, Iaroslav Skiba, David Michonneau, Carl Shultz, William J Hogan, Aitana Balaguer-Roselló, Sara Gil-Perotín, Jan Brijs, Paul Chauvet, Maria Gavriilaki, Ioanna Sakellari, Martin Carre, Adriana Octaviana Dulamea, Alina Daniela Tanase, Yves Chalandon, Sylvain Chantepie, Andrea Duminuco, Urpu Salmenniemi, Michael Loschi, Ron Ram, Irene Garcia Cadenas, Gaetana Porto, Massimo Martino, Patrycja Mensah-Glanowska, Sara Butera, Portia Smallbone, Agnieszka Piekarska, Jeffrey K Davies, Jonathan D. Santoro, Hélène Labussière-Wallet, Marta Gonzalez Vicent, Stéphanie Nguyen, Maud D’Aveni, Mehdi Hamadani, Evelyne Willems, Frédéric Baron, Pierre Maquet, Yves Beguin, Sophie Servais.
