# Supplementary material for: CNS manifestations in acute and chronic graft-versus-host disease
Source: Brain. 2024 Oct 23;148(4):1122–33. doi: 10.1093/brain/awae340 (PMC11967822; doi:10.1093/brain/awae340)
Supplement: awae340_Supplementary_Data [file awae340_supplementary_data.zip › brain-2024-00960-File010.pdf]

Supplementary Material

Supplementary Methods

Definitions used to classify the pCNS-GvHD manifestations into neurologic syndromes

- **Limbic encephalitis**: Working memory deficits, seizures, or psychiatric symptoms suggesting involvement of the limbic system, associated with bilateral brain abnormalities on MRI restricted to the medial temporal lobes and either CSF pleocytosis or EEG with epileptic or slow-wave activity involving the temporal lobes.
- **Brainstem encephalitis**: Presence of at least two clinical criteria indicating involvement of the brainstem (decreased level of consciousness, cranial nerves deficits, ataxia or vertigo, dysarthria or dysphagia), associated with either CSF pleocytosis or abnormalities on MRI restricted to the brainstem.
- **Encephalomyelitis**: Multifocal neurological disorder with clinical manifestations and/or MRI lesions showing evidence of both brain and spinal cord involvement.
- **Extralimbic encephalitis**: Working memory deficits, altered mental status, or psychiatric symptoms, associated with at least one of the following: new focal CNS findings, seizures not explained by a previously known disorder, CSF pleocytosis, MRI brain lesions; and not fulfilling criteria for limbic encephalitis, brainstem encephalitis, or encephalomyelitis.
- **Myelitis**: Motor weakness and/or sensory loss below a certain level, sometimes associated with bowel and/or bladder dysfunction, and associated with MRI lesions restricted to the spinal cord.
- **Meningitis**: Headache associated with CSF pleocytosis without features of encephalitis (limbic encephalitis, brainstem encephalitis, encephalomyelitis, extralimbic encephalitis) or myelitis.
- **Demyelinating disease with neurologic deficits**: multifocal neurologic deficits suggesting involvement of the brain, the spinal cord, and/or the optic nerves, associated with demyelinating lesions of the white matter on brain and/or spinal cord MRI.
- **CNS angiitis**: stroke or rapidly progressive cognitive dysfunction associated with either brain angiography showing segmental narrowing (beading) of cerebral arteries or brain/leptomeningeal histopathological analyses showing granulomatous angiitis, necrotizing vasculitis, or lymphocytic vasculitis.

Statistical analyses: univariate and multivariate Cox Models

First, the impact of socio-demographic and baseline characteristics on the survival probability was investigated using univariate Cox survival models. Hazard ratio and associated 95% CI were presented.

Variables included:
- Age at the beginning of the symptoms (years)
- Sex at birth (male, female)
- Underlying diseases (myeloid malignancy, lymphoid malignancy, non-malignant disease)
- Transplant origin (mobilized peripheral stem cells, other)
- Type of donor (HLA-matched unrelated, HLA-matched familial, HLA-mismatch unrelated, haploidentical familial)
- Sex mismatch between the donor and the recipient (male donor and female patient, female donor and male patient) (yes, no)
- Onset before or after Day 100 following allo-HSCT or DLI (before, after)
- Extra-neurological GVHD flare or progression during the month prior CNS-GVHD occurrence (yes, no)
- Disorder of consciousness at initial presentation (yes, no)
- Increase WBC count in the CSF (yes, no)
- Pathologic brain or spinal cord MRI (yes, no)

Then, various multivariate Cox survival models were investigated:
- The multivariate Cox survival model including all significant variables in univariate case.
- The multivariate Cox survival model including all variables.
- The multivariate Cox survival model including all variables with a stepwise selection of variables.

The multivariate Cox survival model with the better AIC value (i.e., the lower AIC value) was selected and presented in the results.

- The multivariate Cox survival model including all significant variables in univariate case presented an AIC value equal to 236.00.
- The multivariate Cox survival model including all variables presented an AIC value equal to 214.55.
- The multivariate Cox survival model including all variables with a stepwise selection of variables presented an AIC value equal to 201.98.

Thus, the results of multivariate Cox survival model of the survival probability at 1-year follow-up obtained with stepwise selection of variables was selected as this model presented the lower AIC value.

Supplementary Figures

Supplementary Figure 1: Origin of the patients included in the study.

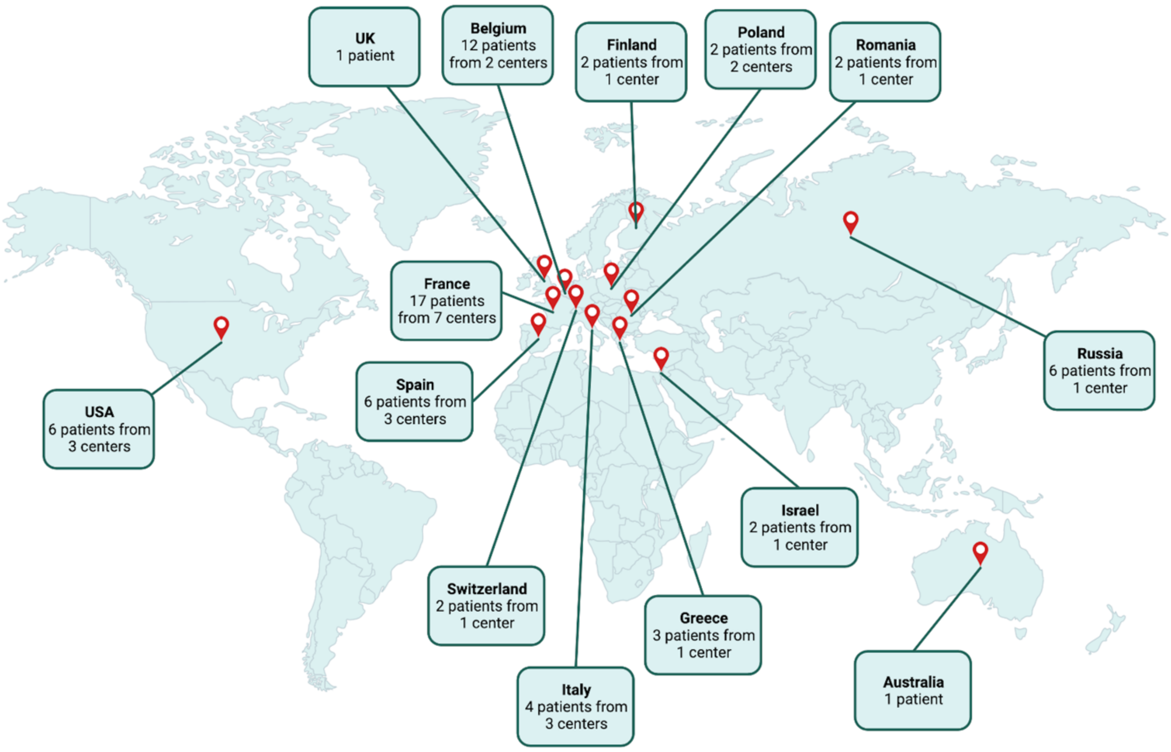

Supplementary Figure 2: Cumulative events curve of cause-specific deaths and cumulative events curve for all death - *N*=56

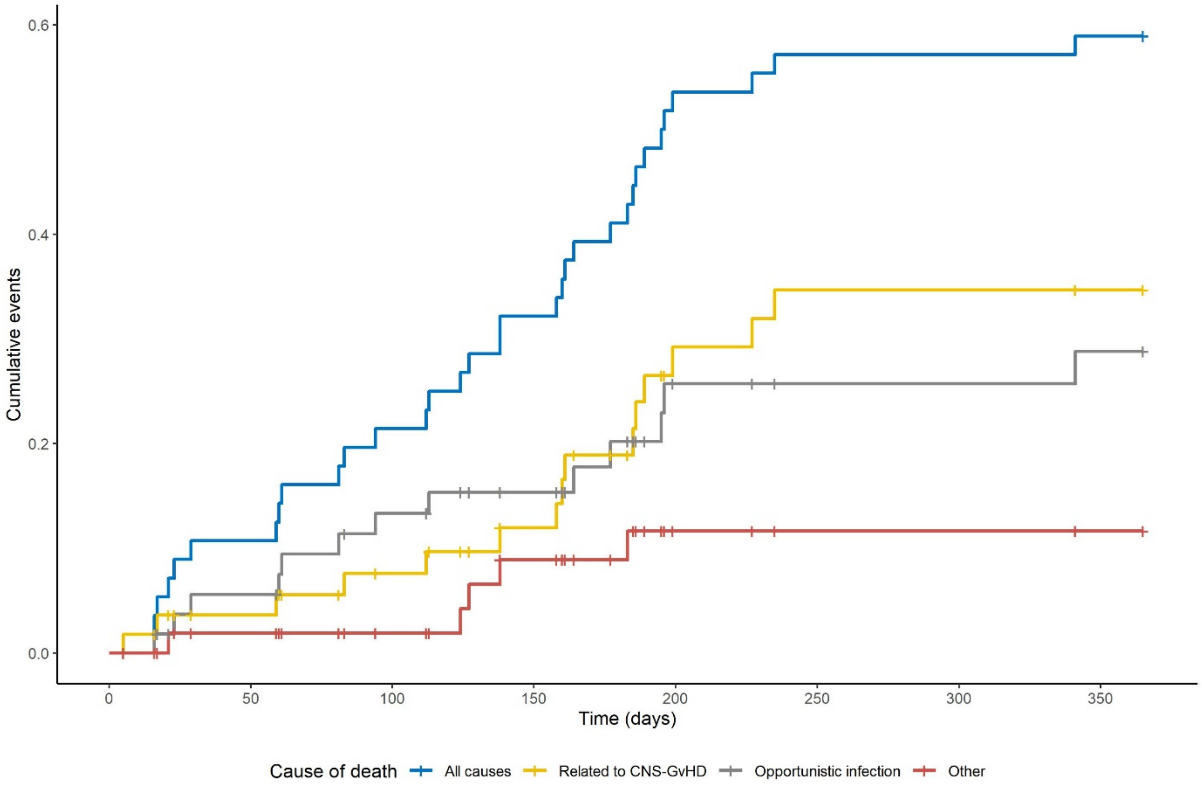

Supplementary Tables

Supplementary Table 1: Characteristics of prior or active extra-neurological acute and chronic GvHD at pCNS-GvHD.

**Characteristics**	**All cases (*N* = 66)**
**GvHD prophylaxis, No. (%)**	
Calcineurin inhibitor + methotrexate	25 (38%)
Calcineurin inhibitor + mycophenolate	36 (55%)
Post-transplant cyclophosphamide	12 (18%)
Anti-T cell globulin	29 (44%)
Other	7 (11%)
**Acute GvHD, No. (% of all cases)**	50 (76%)
Skin	41 (62%)
Gastro-intestinal tract	29 (44%)
Liver	8 (12%)
Multiple organs	24 (36%)
**Acute GvHD grade (MAGIC), No. (% of acute GvHD)**	
I	8 (16%)
II	24 (48%)
III	16 (32%)
IV	2 (4%)
**Chronic GvHD, No. (% of all cases)**	27 (41%)
Skin	19 (29%)
Mouth	13 (20%)
Eyes	9 (14%)
Gastro-intestinal tract	8 (12%)
Liver	5 (8%)
Lungs	4 (6%)
Genital organs	3 (5%)
Muscles or fascia	2 (3%)
Pericardium	1 (2%)
Kidneys	1 (2%)
Peripheral nervous system[b]	9 (14%)
Multiple organs	19 (29%)
**Chronic GvHD grade (NIH), No. (% of chronic GvHD)**	
Mild	9 (33%)
Moderate	9 (33%)
Severe	9 (33%)

GvHD stands for graft-versus-host disease. Acute GVHD grading follows the Mount Sinai Acute GVHD International Consortium (MAGIC) grading system.[4] Chronic GVHD grading follows the National Institute of Health (NIH) scoring system.[5,10]

[a]Other GvHD prophylaxis included tacrolimus without methotrexate or mycophenolate (three patients), corticosteroids (two patients) and sirolimus (one patient).

[b]Seven patients had neuropathy alone, one had myositis, and one had both neuropathy and myasthenic syndrome

Supplementary Table 2: Clinical manifestations of pCNS-GvHD that occurred anytime during the course of the disease.

Clinical signs/symptoms at tany time during the course of the disease	All cases (*N*=66)	pCNS-GvHD ≤100 days (*N*=27)	pCNS-GvHD >100 days (*N*=39)
**Cognitive and/or behavioral impairment, No. (%)**	48 (73%)	23 (85%)	25 (64%)
**Speech impairment, No. (%)**	20 (30%)	8 (30%)	12 (31%)
**Motor impairment, No. (%)**	34 (52%)	12 (44%)	22 (56%)
**One or both upper limb(s)**	22 (33%)	5 (19%)	17 (44%)
**One or both lower limb(s)**	29 (44%)	10 (37%)	19 (49%)
**Gait impairment, No. (%)**	33 (50%)	10 (37%)	23 (59%)
**Vision impairment, No. (%)**	14 (21%)	3 (11%)	11 (28%)
**Sensory impairment, No. (%)**	19 (29%)	5 (19%)	14 (36%)
**Epileptic seizure, No. (%)**	10 (15%)	3 (11%)	7 (18%)
**Headache, No. (%)**	20 (30%)	7 (26%)	13 (33%)
**Hyperkinetic movement disorder, No. (%)**	16 (24%)	7 (26%)	9 (23%)
**Cranial nerve disorder, No. (%)**	9 (14%)	3 (11%)	6 (15%)
**Urinary or anal sphincter dysfunction, No. (%)**	9 (14%)	1 (4%)	8 (21%)
**Disorder of consciousness, No. (%)**	32 (48%)	16 (59%)	16 (41%)

As multiple clinical manifestations may be present, numbers may not sum to group totals, or percentages add to 100%.

Supplementary Table 3: Results of univariate and multivariate binary logistic regressions modelling the response to the treatment depending on different factors.

		Univariate models			Multivariate model (*N*=55)	
**Variable**	**Categories**	***N***	**OR (95% CI)**	***P*-value**	**OR (95% CI)**	***P*-value**
Before or after Day 100 allo-HSCT or DLI (ref.=No)	Yes	64	1.120 (0.321-3.904)	0.86		
Disorder of consciousness at initial presentation (ref.=No)	Yes	64	0.156 (0.039-0.621)	***0.0084***	0.103 (0.022-0.473)	***0.0035***
At least two clinical findings at initial presentation (ref.=No)	Yes	64	0.210 (0.052-0.856)	***0.030***		
WBC count > 5/mm3 in CSF (ref.=No)	Yes	62	1.692 (0.494-5.789)	0.40		
Lesion on MRI (ref.=No)	Yes	63	0.450 (0.110-1.841)	0.27		
Contrast-enhancing lesion on MRI (ref.=No)	Yes	62	0.350 (0.092-1.337)	0.12		
GvHD flair within 30 days prior CNS-GvHD onset (ref.=No)	Yes	59	0.897 (0.261-3.090)	0.86		

Supplementary Table 4: Results of univariate and multivariate Cox survival models of 1-year survival probability in function of baseline characteristics.

		Univariate model			Multivariate model (*N*=48)	
**Variable**	**Categories**	***N***	**HR (95% CI)**	***P*-value**	**HR (95% CI)**	***P*-value**
Age (year)		55	1.02 (0.99-1.05)	0.14		
Sex at birth (ref.= Male)		56		0.97		
	Female		0.98 (0.47-2.07)			
Primary diagnosis (ref.= Non-malignant disease)		56		0.54		
	Lymphoid malignancy		1.39 (0.28-6.87)			
	Myeloid malignancy		1.95 (0.46-8.26)			
Sex mismatch (ref.= No)		55		0.49		
	Yes		0.78 (0.39-1.56)			
Transplant origin (ref.=Other)		56		0.24		
	Mobilized peripheral blood stem cells		1.30 (0.54-3.15)			
Donor type (ref.= HLA-matched familial)		55		0.32		
	HLA-matched unrelated		0.94 (0.35-2.53)			
	HLA-mismatched unrelated		0.15 (0.02-1.31)			
	Haploidentical familial		1.12 (0.35-3.52)			
GVHD flare or progression during the month prior to CNS-GVHD occurrence (ref.= No)		51		0.41		
	Yes		1.33 (0.67-2.63)			
Onset before or after Day 100 following allo-HSCT or DLI (ref.= After)		56		***0.01***		***0.04***
	Before		2.46 (1.23-4.92)		2.14 (1.03-4.45)	
Disorder of consciousness at initial presentation (ref.= No)		56		***0.02***		***0.008***
	Yes		2.52 (1.16-5.45)		2.99 (1.34-6.68)	
Increased WBC count in CSF (ref.= No)		55		0.83		
	Yes		0.93 (0.46-1.86)			
Pathologic MRI (ref.= No)		55		0.05		
	Yes		0.50 (0.25-1.01)			

Supplementary Table 5: Results of univariate cause specific Cox survival model of 1-year survival probability in function of baseline characteristics for death related to pCNS-GvHD.

Variable	Categories	*N*	HR (95% CI)	*P*-value
Age (year)		55	1.029 (0.986-1.074)	0.19
Sex at birth (ref.= Male)		56		0.36
	Female		1.599 (0.585-4.368)	
Primary diagnosis (ref.= Non-malignant disease)		56		0.86
	Lymphoid malignancy		1.225 (0.139-10.798)	
	Myeloid malignancy		1.614 (0.163-15.944)	
Sex mismatch (ref.= No)		55		0.53
	Yes		0.725 (0.268-1.959)	
Transplant origin (ref.=Other)		56		0.49
	Mobilized peripheral stem cells		1.747 (0.364-8.385)	
Donor type (ref.= HLA-matched familial)		48		0.89
	HLA-matched unrelated		1.467 (0.291-7.401)	
	Haploidentical familial		1.299 (0.198-8.498)	
GVHD flare or progression during the month prior CNS-GVHD occurrence (ref.= No)		51		0.41
	Yes		1.521 (0.564-4.105)	
Before or after Day 100 allo-HSCT or DLI (ref.= After)	Before	56		0.49
			0.686 (0.238-1.978)	
Disorder of consciousness at initial presentation (ref.= No)		56		0.25
	Yes		1.954 (0.622-6.142)	
Increased WBC count in CSF (ref.= No)		55		0.67
	Yes		0.803 (0.297-2.173)	
Pathologic MRI (ref.= No)		55		0.99
	Yes		0.992 (0.358-2.747)	

Supplementry Table 6: Results of univariate cause specific Cox survival model of 1-year survival probability in function of baseline characteristics for death related to opportunistic infections.

Variable	Categories	*N*	HR (95% CI)	*P*-value
Age (year)		55	1.008 (0.972-1.045)	0.67
Sex at birth (ref.= Male)		56		0.91
	Female		1.069 (0.330-3.465)	
Primary diagnosis (ref.= Non-malignant disease)		56		0.46
	Lymphoid malignancy		1.572 (0.238-10.377)	
	Myeloid malignancy		0.436 (0.029-6.622)	
Sex mismatch (ref.= No)		55		0.38
	Yes		0.585 (0.191-1.795)	
Transplant origin (ref.=Other)		56		0.95
	Mobilized peripheral stem cells		0.606 (0.197-1.860)	
Donor type (ref.= HLA-matched familial)		48		0.95
	HLA-matched unrelated		1.003 (0.193-5.210)	
	Haploidentical familial		1.216 (0.199-7.428)	
GVHD flare or progression during the month prior CNS-GVHD occurrence (ref.= No)		51		0.60
	Yes		0.747 (0.248-2.249)	
Before or after Day 100 allo-HSCT or DLI (ref.= After)	Before	56		*0.023*
			3.831 (1.202-12.212)	
Disorder of consciousness at initial presentation (ref.= No)		56		0.15
	Yes		2.311 (0.734-7.273)	
Increased WBC count in CSF (ref.= No)		55		0.72
	Yes		0.820 (0.281-2.390)	
Pathologic MRI (ref.= No)		55		0.16
	Yes		0.440 (0.142-1.370)	

Due to the low number of events, no multivariate model was performed.

Supplementry Table 7: Patient and transplant-related characteristics of patients with possible chronic CNS-GvHD following 2010 Consensus Conference on Clinical Practice in Chronic GvHD criteria.

**Patients and transplant characteristics**	**Patients (n = 27)**
Male sex, No. (%)	21 (78%)
Age at pCNS-GvHD (years), median ($IQ_{25–75}$)	56 (34–66)
Underlying disease, No. (%)	
Myeloid malignancies	17 (63%)
Lymphoid malignancies	6 (22%)
Non-malignant diseases	4 (15%)
Non-hematological disorder prior to allo-HSCT	
CNS disorder, No. (%)	6 (22%)
Immune-mediated disorder, No. (%)	3 (11%)
Conditioning regimen before allo-HSCT	
Myeloablative, No. (%)	12 (44%)
TBI-based, No. (%)	10 (37%)
Source of stem cells, No. (%)	
Mobilized peripheral blood stem cells	20 (74%)
Bone marrow	5 (19%)
Cord blood	2 (7 %)
Donor type, No. (%)	
Related, HLA-matched	5 (19%)
Related, HLA-haploidentical	5 (19%)
Unrelated, HLA-matched	13 (48%)
Unrelated, HLA-mismatched	4 (15%)
Donor-recipient sex mismatch (female for male), No. (%)	12 (44%)
GvHD prophylaxis, No. (%)	
Calcineurin inhibitor + methotrexate	8 (30%)
Calcineurin inhibitor + mycophenolate	17 (63%)
Post-transplant cyclophosphamide	3 (11%)
Anti-T cell globulin	10 (37%)
Other	3 (11%)
Donor lymphocyte infusion before pCNS-GvHD, No. (%)	1 (4%)
Extra-CNS aGvHD prior or concomitant to pCNS-GvH D, No. (%)	19 (70%)
Delay between allo-HSCT/ DLI and pCNS-GvHD (days), median ($IQ_{25–75}$)	290 (236–408)

Allo-HSCT stands for allogeneic hematopoietic stem cell transplantation, aGvHD for acute graft-versus-host disease, DLI for donor lymphocyte infusion, and pCNS-GvHD for possible central nervous system graft-versus-host disease.

Supplementary Table 8: Clinical manifestations of possible chronic CNS-GvHD following the 2010 Consensus Conference on Clinical Practice in Chronic GvHD criteria and neurological sequelae among surviving patients one year after pCNS-GvHD onset.

Clinical sign/symptom	Patients
**At initial presentation**	***N*=27**
Cognitive and/or behavioral impairment, No. (%)	7 (26%)
Speech impairment, No. (%)	2 (7%)
Motor impairment, No. (%)	5 (19%)
One or both upper limb(s)	1 (4%)
One or both lower limb(s)	5 (19%)
Gait impairment, No. (%)	4 (15%)
Vision impairment, No. (%)	6 (22%)
Sensory impairment, No. (%)	7 (26%)
Epileptic seizure, No. (%)	2 (7%)
Headache, No. (%)	4 (15%)
Hyperkinetic movement disorder, No. (%)	5 (19%)
Cranial nerve disorder, No. (%)	2 (7%)
Urinary or anal sphincter dysfunction, No. (%)	1 (4%)
Disorder of consciousness, No. (%)	4 (15%)
**At any time during the course of the disease**	***N*=27**
Cognitive and/or behavioral impairment, No. (%)	19 (70%)
Speech impairment, No. (%)	9 (33%)
Motor impairment, No. (%)	14 (52%)
One or both upper limb(s)	9 (33%)
One or both lower limb(s)	13 (49%)
Gait impairment, No. (%)	13 (48%)
Vision impairment, No. (%)	9 (33%)
Sensory impairment, No. (%)	10 (37%)
Epileptic seizure, No. (%)	4 (15%)
Headache, No. (%)	10 (37%)
Hyperkinetic movement disorder, No. (%)	10 (37%)
Cranial nerve disorder, No. (%)	4 (15%)
Urinary or anal sphincter dysfunction, No. (%)	4 (15%)
Disorder of consciousness, No. (%)	12 (44%)
**Neurological sequelae one year after disease onset**	***N*=11**
Cognitive and/or behavioral impairment, No. (%)	3 (27%)
Speech impairment, No. (%)	0 (0%)
Motor impairment, No. (%)	1 (9%)
One or both upper limb(s)	1 (9%)
One or both lower limb(s)	1 (9%)

Neurological sequelae one year after disease onset (continued)	
Gait impairment, No. (%)	4 (36%)
Vision impairment, No. (%)	1 (9%)
Sensory impairment, No. (%)	1 (9%)
Epileptic seizure, No. (%)	0 (0%)
Headache, No. (%)	1 (9%)
Hyperkinetic movement disorder, No. (%)	1 (9%)
Cranial nerve disorder, No. (%)	1 (9%)
Urinary or anal sphincter dysfunction, No. (%)	2 (18%)
Disorder of consciousness, No. (%)	1 (9%)
No clinical neurological sequelae, No. (%)	3 (27%)

Supplementary Table 9: Biological and radiological characteristics of patients diagnosed with possible chronic CNS-GvHD following the 2010 Consensus Conference on Clinical Practice in Chronic GvHD criteria.

**MRI and CSF characteristics**	**Patients**
Patients with brain MRI results available, No.	27
Brain lesions seen with MRI compatible with symptomatology, No. (%)	13 (54%)
Among these,	
Supratentorial lesions, No. (%)	12 (86%)
Infratentorial lesions, No. (%)	5 (43%)
Contrast-enhancing lesions, No. (%)	3 (34%)
Multiple lesions, No. (%)	12 (83%)
Type of lesions, No. (%)	
Separate oval or punctuate white matter lesions	8 (62%)
Confluent white matter lesions	5 (38%)
Acute ischemic lesions	0 (0%)
Pseudo-tumoral lesions	0 (0%)
Extra-parenchymal intracranial lesions, No. (%)	2 (5%)
Patients with spinal cord MRI results available, No.	14
Spinal cord lesions seen with MRI, No. (%)	4 (29%)
Among these	
Longitudinally extensive, No. (%)	4 (100%)
Contrast-enhancing lesions, No. (%)	3 (75%)
Multiple lesions, No. (%)	3 (75%)
Patients with brain CT results available, No.	16
Brain lesions seen with CT compatible with symptomatology, No. (%)	1 (6%)
Patients with CSF results available, No.	27
CSF WBC count > 5/mm^3, No. (%)	14 (52%)
Among these, WBC count (cells/mm^3), median ($IQ_{25–75}$)	30 (12-46)
CSF protein level > 0.45 g/L, No. (%)	22 (81%)
CSF protein level (g/L), median ($IQ_{25–75}$)	0.8 (0.52-1.29)
CSF glucose level < 0.45 mg/dL, No. (%)	1 (4%[a])
CSF glucose level (mg/dL), median ($IQ_{25–75}$)	57.5 (53-70)
CSF oligoclonal bands, No. (%)	10 (67%)

CSF stands for cerebrospinal fluid, CT for computed tomography, MRI for magnetic resonance imaging, WBC for white blood cell.

[a]Data on CSF oligoclonal bands was available for 15 patients.

Supplementary Table 10: Treatments administrated as first-line regimen among patients with possible chronic CNS-GvHD following 2010 Consensus Conference on Clinical Practice in Chronic GvHD criteria, response to treatment, relapse rate, and one-year overall survival.

**First-line therapy**	**Patients (*N*=27)**
Corticosteroids, No. (%)	23 (85%)
Calcineurin inhibitor, No. (%)	5 (19%)
Mycophenolate mofetil, No. (%)	3 (11%)
Intravenous immunoglobulins, No. (%)	6 (22%)
Plasma exchanges, No. (%)	4 (15%)
Rituximab, No. (%)	2 (7%)
Cyclophosphamide, No. (%)	2 (7%)
Ruxolitinib, No. (%)	3 (11%)
Tocilizumab, No. (%)	0 (0%)
Fingolimod, No. (%)	0 (0%)
Sirolimus, No. (%)	1 (4%)
Combination of at least two treatments, No. (%)	15 (56%)
**Clinical response to treatment**	**Patients (*N*=27)**
Complete clinical, No. (%)	7 (26%)
Partial improvement, No. (%)	14 (52%)
Stabilization of the disease, No. (%)	2 (7%)
No response, No. (%)	4 (15%)
**Relapse rate among patients who responded to treatment**	**Patients (*N*=23)**
Relapse, No. (%)	10 (43%)
**One-year overall survival**	**Patients (*N*=24)**
Survival, No. (%)	11 (46%)

Supplementary Table 11: Comparison of the clinical, biological, and radiological characteristics, and of the relapse rate between patients with pCNS-GvHD meeting the 2010 criteria for possible chronic CNS-GvHD and patients with pCNS-GvHD occurring more than 100 days following allo-HSCT and not meeting 2010 criteria.

Clinical sign/symptom	Patients with chronic pCNS-GvHD meeting 2010 criteria (*N*=27)	Patients with chronic pCNS-GvHD not meeting 2010 criteria* (*N*=20)	Comparison (*P* value)
**Clinical characteristics**			
Cognitive and/or behavioral impairment, No. (%)	19 (70%)	13 (65%)	0.76
Speech impairment, No. (%)	9 (33%)	6 (30%)	>0.99
Motor impairment, No. (%)	14 (52%)	12 (60%)	0.77
Gait impairment, No. (%)	13 (48%)	12 (60%)	0.56
Vision impairment, No. (%)	9 (33%)	3 (15%)	0.19
Sensory impairment, No. (%)	10 (37%)	5 (25%)	0.53
Epileptic seizure, No. (%)	4 (15%)	4 (20%)	0.76
Headache, No. (%)	10 (37%)	6 (30%)	>0.99
Hyperkinetic movement disorder, No. (%)	10 (37%)	3 (15%)	0.11
Cranial nerve disorder, No. (%)	4 (15%)	2 (10%)	>0.99
Urinary or anal sphincter dysfunction, No. (%)	4 (15%)	4 (20%)	0.76
Disorder of consciousness, No. (%)	12 (44%)	8 (40%)	>0.99
**Radiological and CSF characteristics**			
Brain or spinal cord lesions seen with MRI compatible with symptomatology, No. (%)	17 (63%)	15 (75%)	0.53
Increased white blood cell count (>5/mm^3) in the CSF, No. (%)	14 (52%)	13 (65%)	0.39
**Relapse**			
Number of patients who responded to treatment, No.	23	14	
Relapse rate among patients with a clinical response to first-line immunosuppressive therapy, No. (%)	10 (43%)	5 (36%)	0.74

*As 2010 criteria only allow the diagnosis of chronic CNS-GvHD, only patients with pCNS-GvHD >100 days not meeting 2010 criteria were included in the other group for comparative analyses.

Supplementary Table 12: Distribution of response to the treatment and death at 1-year of follow-up depending on whether patients with chronic pCNS-GvHD met 2010 diagnosis criteria.

				Meeting 2010 Criteria				
		All		No		Yes		
**Variable**	**Categories**	***N***	**Number (%)**	***N***	**Number (%)**	***N***	**Number (%)**	***P*-value**
Response to the treatment		46		19		27		0.34[a]
	No		9 (19.6)		5 (26.3)		4 (14.8)	
	Yes		37 (80.4)		14 (73.7)		23 (85.2)	
Alive at 1-year follow-up		39		15		24		0.43[b]
	No		20 (51.3)		7 (46.7)		13 (54.2)	
	Yes		19 (48.7)		8 (53.3)		11 (45.8)	

a: binary logistic regression p-value

b: log-rank test p-value

As 2010 criteria only allow the diagnosis of chronic CNS-GvHD, only patients with pCNS-GvHD >100 days not meeting 2010 criteria were included in the other group for comparative analyses.

Supplementary Table 13: Distribution of neurological syndromes depending on the status (survival or death) after one year of follow-up

				Alive at 1-year follow-up				
		All		No		Yes		
**Variable**	**Categories**	**N**	**Number**	**N**	**Number (%)**	**N**	**Number (%)**	***P*-value**
Neurological syndrome		56		33		23		0.30[a]
	Brainstem encephalitis		4		3 (75)		1 (25)	
	CNS angiitis		1		1 (100)		0 (0)	
	Demyelinating disease with neurologic deficits		7		3 (43)		4 (57)	
	Encephalomyelitis		4		2 (50)		2 (50)	
	Extralimbic encephalitis		35		23 (66)		12 (34)	
	Meningitis		2		1 (50)		1 (50)	
	Myelitis		3		0 (0)		3 (100)	

a: Due to the low number of subjects per group, Fisher's exact tests was used.

Supplementary Table 14: Distribution of neurological syndromes depending on the response to the treatment

				Response to the treatment				
		All		No		Yes		
**Variable**	**Categories**	**N**	**Number**	**N**	**Number (%)**	**N**	**Number (%)**	***P*-value**
Neurological syndrome		64		13		51		0.68[a]
	Brainstem encephalitis		4		0 (0)		4 (100)	
	CNS angiitis		1		0 (0)		1 (100)	
	Demyelinating disease with neurologic deficits		9		3 (33)		6 (67)	
	Encephalomyelitis		5		2 (40)		3 (60)	
	Extralimbic encephalitis		40		8 (20)		32 (80)	
	Meningitis		2		0 (0)		2 (100)	
	Myelitis		3		0 (0)		3 (100)	

a: Due to the low number of subjects per group, Fisher's exact tests was used.

Supplementary Table 15: Distribution of neurological syndromes depending on the delay between allo-HSCT or DLI and the onset of pCNS-GvHD

				Before or after Day 100 following allo-HSCT or DLI				
		All		After		Before		
**Variable**	**Categories**	**N**	**Number**	**N**	**Number (%)**	**N**	**Number (%)**	***P*-value**
Neurological syndrome		66		39		27		0.25[a]
	Brainstem encephalitis		4		1 (25)		3 (75)	
	CNS angiitis		1		1 (100)		0 (0)	
	Demyelinating disease with neurologic deficits		9		7 (78)		2 (22)	
	Encephalomyelitis		5		4 (80)		1 (20)	
	Extralimbic encephalitis		42		22 (52)		20 (48)	
	Meningitis		2		1 (50)		1 (50)	
	Myelitis		3		3 (100)		0 (0)	

a: Due to the low number of subjects per group, Fisher's exact tests was used.
