# Supplementary material for: CNS manifestations in acute and chronic graft-versus-host disease
Source: Brain. 2024 Oct 23;148(4):1122–33. doi: 10.1093/brain/awae340 (PMC11967822; doi:10.1093/brain/awae340)
Supplement: awae340_Supplementary_Data [file awae340_supplementary_data.zip › brain-2024-00960-File011.pdf]

CNS-GvHD Study Group members and affiliations

Nicolas Lambert	Department of Neurology, University Hospital of Liège, Liège, Belgium
Florence Forte	Department of Neurology, University Hospital of Liège, Liège, Belgium
Majdouline El Moussaoui	Department of Infectious diseases and General Internal Medicine, University Hospital of Liège, Liège, Belgium
Justine Monseur	Biostatistics and Research Methods center (B-STAT), Department of Public Health, University of Liège, Liège, Belgium
Nicole Raus	Department of Hematology Marcel-Bérard secteur 1G, Centre hospitalier Lyon sud, 69310 Pierre-Bénite, France.
Alexey Polushin	Department of Chemotherapy and Stem Cell Transplantation for Cancer and Autoimmune Diseases, First Pavlov State Medical University of St. Peterburg, 197022 Saint Petersburg, Russia
Iaroslav Skiba	Department of Chemotherapy and Stem Cell Transplantation for Cancer and Autoimmune Diseases, First Pavlov State Medical University of St. Peterburg, 197022 Saint Petersburg, Russia
David Michonneau	Hematology and transplantation unit, Saint Louis hospital, Université Paris Cité, Paris, France
Carl Shultz	Division of Hematology, Mayo Clinic, Rochester, Minnesota, USA
William J Hogan	Division of Hematology, Mayo Clinic, Rochester, Minnesota, USA
Aitana Balaguer-Roselló	Department of Hematology, Hospital Universitari i Politècnic la Fe, Valencia, Spain
Sara Gil-Perotín	Department of Neurology, Hospital Universitari i Politècnic la Fe, Valencia, Spain
Jan Brijs	Department of Hematology, University Hospitals Leuven, Leuven, Belgium
Paul Chauvet	CHU de Lille, Maladies du Sang, Université de Lille, 59000, Lille, France
Maria Gavriilaki	First Department of Neurology, AHEPA University Hospital, Aristotle University of Thessaloniki, Greece
Ioanna Sakellari	BMT Unit - Department of Hematology, G. Papanicolaou Hospital, Thessaloniki, Greece
Martin Carre	Department of Hematology, Hôpital Michallon, Grenoble, France
Adriana Octaviana Dulamea	Department of Neurology, University of Medicine and Pharmacy “Carol Davila”, Fundeni Clinical Institute, Bucharest, Romania
Alina Daniela Tanase	Department of Bone Marrow Transplantation, Fundeni Clinical Institute, Bucharest, Romania
Yves Chalandon	Division of Hematology, University Hospital of Geneva (HUG) and faculty of Medicine, University of Geneva, Geneva, Switzerland
Sylvain Chantepie	Department of Hematology, University hospital of Caen, Caen, France
Andrea Duminuco	Department of Hematology with BMT, A.O.U. Policlinico “G.Rodolico-San Marco”, Catania, Italy
Urpu Salmenniemi	HUCH Comprehensive Cancer Center, Stem Cell Transplantation Unit - Helsinki, Helsinki, Finland
Michael Loschi	Department of Hematology, University Hospital of Nice, Cote d’Azur University, Nice, France
Ron Ram	BMT Unit, Tel Aviv Sourasky Medical Center and Faculty of Medicine, Tel Aviv University, Tel Aviv, Israel
Irene Garcia Cadenas	Department of Hematology. Hospital de la Santa Creu i Sant Pau. Barcelona, Spain
Gaetana Porto	Department of Hemato-Oncology and Radiotherapy, Stem Cell Transplantation and Cellular Therapies Unit (CTMO), Grande Ospedale Metropolitano "Bianchi-Melacrino-Morelli", Reggio Calabria, Italy
Massimo Martino	Stem Cell Transplant and Cellular Therapies Unit, Great Metropolitan Hospital "Bianchi-Melacrino-Morelli", 89133 Reggio Calabria, Italy
Patrycja Mensah-Glanowska	Department of Hematology, Jagiellonian University Medical College, University Hospital in Krakow, Krakow, Poland
Sara Butera	Department of Hematology, AO SS Antonio e Biagio E Cesare Arrigo, Alessandria, Italy
Portia Smallbone	Department of Hematology, Fiona Stanley Hospital, Perth, Western Australia

Agnieszka Piekarska	Department of Hematology and Transplantology, University Clinical Center, Medical University of Gdańsk, Gdańsk, Poland
Jeffrey K Davies	Centre for Haemato-Oncology, Bart Cancer Institute, Queen Mary University of London and Barts Health NHS Trust, London, UK
Jonathan D. Santoro	Division of Neurology, Department of Pediatrics, Children’s Hospital Los Angeles, Los Angeles, California, USA
Hélène Labussière-Wallet	Hôpital Lyon Sud, Pierre Bénite, France
Marta Gonzalez Vicent	BMT Unit, Hospital Niño Jesus, Madrid, Spain
Stéphanie Nguyen	Department of Hematology, Pitie-Salpetriere Hospital, 75013 Paris, France Société Francophone de Greffe de Moelle et de Thérapie Cellulaire
Maud D’Aveni	Department of Clinical Hematology, Centre Hospitalier Régional Universitaire de Nancy, Vandoeuvre-lès-Nancy, France
Mehdi Hamadani	Department of Medicine, Medical College of Wisconsin, Milwaukee, USA
Evelyne Willems	Department of Hematology, University Hospital of Liège, Liège, Belgium
Frédéric Baron	Department of Hematology, University Hospital of Liège, Liège, Belgium
Pierre Maquet	Department of Neurology, University Hospital of Liège, Liège, Belgium
Yves Beguin	Department of Hematology, University Hospital of Liège, Liège, Belgium
Sophie Servais	Department of Hematology, University Hospital of Liège, Liège, Belgium
